# Relationships between nursing diagnoses and the level of dependence in activities of daily living of elderly residents

**DOI:** 10.31744/einstein_journal/2020AO5445

**Published:** 2020-10-29

**Authors:** Karina Mello Dias, Tracy Heather Herdman, Renata Eloah de Lucena Ferretti-Rebustini, Camila Takao Lopes, Eduarda Ribeiro dos Santos

**Affiliations:** 1 Faculdade Israelita de Ciências da Saúde Albert Einstein Hospital Israelita Albert Einstein São PauloSP Brazil Faculdade Israelita de Ciências da Saúde Albert Einstein, Hospital Israelita Albert Einstein, São Paulo, SP, Brazil.; 2 University of Wisconsin-Green Bay Green BayWI United States University of Wisconsin-Green Bay, Green Bay, WI, United States.; 3 Universidade de São Paulo Escola de Enfermagem São PauloSP Brazil Escola de Enfermagem, Universidade de São Paulo, São Paulo, SP, Brazil.; 4 Universidade Federal de São Paulo Escola Paulista de Enfermagem São PauloSP Brazil Escola Paulista de Enfermagem, Universidade Federal de São Paulo, São Paulo, SP, Brazil.

**Keywords:** Aged, Geriatric nursing, Homes for the aged, Nursing diagnosis, Nursing processes

## Abstract

**Objective:**

To identify and validate nursing diagnoses of elderly residents, and determine their relationship with the level of dependence in activities of daily living.

**Methods:**

One hundred thirty-five older adults were assessed using medical history and physical examination. Twelve validated gerontological instruments were administered to assess delirium, nutritional status, risk for falls, risk for pressure injury, dementia, cognitive losses, depression, and level of dependence in daily living and instrumental activities of daily living. Nursing diagnoses were identified and validated by experienced, doctorally-prepared nurses. The association between the presence of a nursing diagnosis and the level of dependence was assessed by a test for trend in proportions. The Kruskal-Wallis hypothesis test was used to investigate the association between the number of nursing diagnoses and the level of dependence of the elderly.

**Results:**

Most older adults were at risk for malnutrition, at high risk for falls, cognitively impaired, totally dependent for daily living and activities of daily living. In addition, they had very mild dementia and most did not have risk for pressure injuries. Depression was noted among those with dementia, but was absent in those without dementia. A total of 52 nursing diagnoses were validated. Of these, 11 were associated with the level of dependence in daily living.

**Conclusion:**

These results can be reproduced in other skilled nursing facilities for older adults, and these may allow the planning of interventions to alleviate etiologies and signs/symptoms of nursing diagnoses, rather than simply directing care toward a general category of dependence. Therefore, guaranteeing individualized nursing care to meet the specific needs of each resident.

## INTRODUCTION

Worldwide, population aging has been occurring rapidly in the last decades. In 2020, the *Instituto Brasileiro de Geografia e Estatística* (IBGE) estimates that there are 20,816 million elderly people in Brazil, which is expected to double by 2044. Additionally, it is anticipated that the elderly population will account for more than 25% of the total population in Brazil by 2060, surpassing the indicators estimated for developed countries.^(^^1^^)^

Despite technological advances, which aid in disease prevention and treatment, the elderly population is marked by reduced functional capacities, leading to a variety of physical and psychological limitations, difficulty performing daily activities, and maintaining acceptable social behavior.^(^^2^^)^

Functional decline refers to the difficulty, or need for help, performing daily activities, including activities of daily living (ADL) and instrumental ADL (IADL). In gerontology, assessment of functionality of elderly individuals is an important parameter of ADL, as it provides relevant information about their health and about the need for support in performing ADL.^(^^2^^)^

Due to the extended lives of the population, there is often the need for settings with adequate structures to care for these individuals, such as nursing home settings for the elderly. In these institutions, a specialized multidisciplinary team is required. Nurses play a leading role in the care, education and teaching of residents, with a direct impact on the quality of care.^(^^3^^,^^4^^)^

From the nursing perspective, identifying accurate nursing diagnoses (NDx) is essential in order to establish patient-centered outcomes, and to plan appropriate interventions. The use of validated measurement instruments is recommended by NANDA International, Inc., (NANDA-I) to ensure accurate, relevant NDx.^(^^4^^)^

A few older studies on the nursing process in skilled nursing facilities in Brazil were identified in the literature, however none of these validated their findings with regard to accuracy through the use of validated gerontological scales.^(^^5^^,^^6^^)^ Therefore, the aims of this study were to identify and validate NDx in elderly nursing home residents, and to determine the relationship between the NDx and the level of dependence of these residents.

## OBJECTIVE

To identify and validate nursing diagnoses in elderly nursing home residents, and to determine the relationship between the nursing diagnoses and the level of dependence of these residents.

## METHODS

### Design and setting

An analytical, cross-sectional study performed at *Residencial Israelita Albert Einstein* (RIAE), a nursing home for the elderly, in the city of São Paulo (SP), Brazil, with the capacity to care for 170 elderly residents.

### Participants

A convenience sample of 135 individuals residing in the institution, regardless of gender or age, between May and July of 2016.

### Data collection

A geriatric nurse specialist collected data on the medical history and physical examination of the residents. In addition, the nurse used 12 validated gerontological instruments to investigate delirium, nutritional status, risk for falls, risk for pressure injury, dementia, cognitive status, depression, and level of dependence in ADL and IADL. Table 1 identifies all scales, the focus of their measurement, who conducted the assessment, from whom data was obtained, characteristics considered by the tools, how they are scored, and critical references.

**Table 1:** Geriatric assessment tools used to evaluate the residents

Scale Name	Focus of assessment	Who conducted assessment	From whom data was obtained	Characteristics considered	Scoring
Confusion Assessment Method for Intensive Care Unit^(^^7^^)^	Delirium	Nurse researcher	Resident	1. Acute onset and fluctuating course of delirium symptoms 2. Inattention 3. Altered level of consciousness 4. Disorganized thinking	When features 1 and 2 are both present and either features 3 or 4 are present: Confusion Assessment Method for Intensive Care Unit is positive, delirium is present
Mini Nutritional Assessment^®(8)^	Nutritional status	Nurse researcher	Resident	18 items encompassing anthropometry, dietary assessment, global clinical evaluation and self-perception of health and nutritional status	24-30 points: normal nutritional status 17-23.5 points: at risk of malnutrition 17 points: malnourished
Downton Fall Risk Index^(^^9^^)^	Fall risk	Nurse researcher	Resident, medical records and family members	Past history of falls, medications, sensory *deficits*, mental state, and walking activities	Each factor can obtain a score of 1 point, with scores ≥3 identifying patients at risk
Braden scale^(^^10^^)^	Pressure injury risk	Nurse researcher	Resident	6 subscales, namely: sensory perception, activity, mobility, moisture, nutrition, friction or shear	Total score can vary from 6-23 points, and the patients are classified as follows: very high risk of pressure injury (≤9 points), high risk (10-12), moderate risk (13-14), low risk (15-18) and no risk (19-23)
Clinical Dementia Rating^(^^11^^)^	Cognitive and functional performance in dementia	Researcher and a physician	Resident and/or family member	6 domains: memory, orientation, judgment & problem solving, community affairs, home & hobbies, and personal care	Five-point scale. An overall score is calculated through the use of an algorithm – 0: normal – 0.5: very mild dementia – 1: mild dementia – 2: moderate dementia – 3: severe dementia
Mini-Mental State Examination^(^^12^^)^	Cognitive impairment	Nurse researcher	Resident	30-point questionnaire grouped into 7 categories, each representing a different cognitive domain/function: orientation to time (5 points), orientation to place (5 points), registration of 3 words (3 points), attention and calculation (5 points), recall of 3 words (3 points), language (8 points) and visual construction (1 point)	The cutoff points for cognitive impairment vay according to the level of education: – 18: illiterate – 21: 1 to 3 years of education – 24: 4 to 7 years of education – 26: over 7 years of education
Semantic Verbal Fluency^(^^13^^)^	Cognitive impairment	Nurse researcher	Resident	Tracks the storage capacity of the semantic memory system, the ability to retrieve information stored in memory, and the processing of executive functions. This test includes reporting as many animal names as possible within a 1-minute period, and assigns 1 point per animal named	Results are scored as follows: 9 points: illiterate; 12 points: 1 to 8 years of study; 13 points: 9 years or more of study.
Clock Drawing Test^(^^14^^)^	Cognitive impairment	Nurse researcher	Resident	Residents were given a blank sheet of paper and a pen, and were then asked to follow the instructions: “First, draw a clock with all the numbers on it. Second, put hands on the clock to make it read 2:45”	10-6 points: drawing of clock face with circle and numbers is generally intact 5-1 point: drawing of clock face with circle and numbers is not intact Scores <6 are classified as cognitive impairment
Geriatric Depression Scale: Short Form^(^^15^^)^	Depression in elderly without dementia	Nurse researcher	Resident	15-item scale, in which 10 items indicate the presence of depression when answered positively, whereas 5 items indicate depression when answered negatively	− 0-5: absence of depression – 6-10: mild depression – 9-11: moderate depression – 11-15: severe depression
Cornell Scale^(^^16^^)^	Depression in elderly with dementia	Nurse researcher	Caregiver interview	19 items, each of which is evaluated for severity, on a scale of 0-2: – 0: absent – 1: slight or intermittent – 2: severe	Total score ≤9 equates to absence of depression; 10-17 indicates probable depression; 18 correlates to depression
Katz Index^(^^17^^)^	Autonomy in ADL	Nurse researcher	Resident, medical records and family member	Assess by ranking adequacy of performance in bathing, dressing, toileting, transferring, continence, and feeding. In each activity, the residents were scored “yes” or “no” for independence	Classified as totally dependent when ≤2 yes scores were obtained; partially dependent (3 or 4 points); totally dependent (5 or 6 points)
Lawton & Brody Scale^(^^18^^)^	Independence in IADL	Nurse researcher	Resident and family member	8 domains: ability to use telephone, shopping, food preparation, housekeeping, laundry, mode of transportation, responsibility for own medications and ability to handle finances	- Score of 0-1: total dependence – 2-3: severe dependence – 4-5: moderate dependence – 6-7: mild dependence – 8: independent

ADL: activities of daily living; IADL: instrumental activities of daily living.

After data collection, NANDA-I NDx hypotheses were identified by a geriatric nurse specialist and a doctorally prepared nurse. Next, the identified NDx were assessed by three clinically experienced, doctorally prepared nurses, with scientific publications related to gerontology and the Nursing Process.

The main investigator previously emailed a file to the experts, containing data collection from each resident. The experts met face-to-face three times, for approximately 4 hours, reviewed the description of each patient, and discussed each nursing diagnosis for each resident, until a 100% consensus was achieved on the relevance of the NDx supported by the NANDA-I diagnostic classification, 2015-2017.^(^^19^^)^

### Ethical considerations

The study participants or their family members signed Consent Forms, ensuring their anonymity, as well as guaranteeing the freedom not to participate or to withdraw from the research, if they so wished. The research protocol was approved by the institutional Research Ethics Committee, protocol no. 1.446.621, CAAE: 53671316.4.0000.0071.

### Data analysis

The NDx were analyzed descriptively (absolute and relative frequencies) according to the Katz Index level of dependence in ADL. The association between the presence of a NDx and the level of dependence was assessed by a test for trend in proportions. In order to investigate the association between the number of diagnoses and the level of dependence of the elderly, the Kruskal-Wallis hypothesis test was used. The analyses were performed using R software, or SPSS software version 3.1.3. A significance level of 5% was considered.

## RESULTS

Table 2 shows the clinical and demographic profile of the residents. All were Jewish, aged 60 to 102 years, most were female, widowed, and were housewives before living in the institution. They slept a satisfactory number of hours per night (7.65±1.31 hours), although 75.6% (n=102) used medication to induce sleep. Smoking or alcohol use were not common. Most had a visual *deficit* or unsteady gait, and required mobility devices. The mean body mass index (BMI) was 27.5±5.5kg/m^2^. Forty-three (31.9%) were obese, 48 (35.6%) were overweight, 41 (30.4%) had a normal weight and 3 (2.22%) were underweight. The most common feeding method was oral, with good food acceptance. Of those fed orally, 20 (15.9%) required the use of thickeners. Thirty-six (26.7%) used supplements. The mean fluid intake was 810±350mL/day.

**Table 2:** Clinical and sociodemographic characteristics of the elderly residents

Variables		Mean±SD
Age (years old)		85.7±8.0
		n (%)
Gender		
	Female	107 (79.3)
	Male	28 (20.7)
Religion: Judaism		135 (100.0)
Marital status		
	Widow(er)	81 (60.0)
	Single	28 (20.7)
	Married	20 (14.8)
	Divorced	6 (4.4)
Occupation before living in the institution		
	Housewife	59 (43.7)
	Tradesman/Tradewoman	30 (22.2)
	Teacher	9 (6.7)
	Secretary	9 (6.7)
	Other	28 (20.7)
Habits		
	Smoking	3 (2.2)
	Alcohol use	2 (1.5)
	Use of sleeping pills	102 (75.6)
Feeding method		
	Oral	126 (93.3)
	Gastrostomy	9 (6.7)
Food acceptance		
	Good	109 (86.5)
	Fair	15 (11.9)
	Bad	2 (1.6)
Use of oral supplements		36 (26.7)
Visual *deficit*		102 (75.6)
Hearing loss		56 (41.5)
Speech impairment		4 (3.0)
Unsteady gait		103 (76.3)
Mobility device requirement		
	Wheelchair	64 (45.9)
	None	34 (26.7)
	Walker	33 (24.4)
	Cane	4 (3.0)

SD: standard deviation.

Table 3 shows clinical assessments based on the validated geriatric instruments. Most residents were at risk of malnutrition, had a high risk for falls, did not have a risk for pressure injuries, very mild dementia, had cognitive impairment, depression among those with dementia, absence of depression among those without dementia, total dependence in ADL and IADL.

**Table 3:** Clinical assessment of the elderly residents through validated instruments

Assessment		n (%)
Nutritional status according to the Mini Nutritional Assessment^®^ (n=135)		
	Malnourished	25 (18.5)
	At risk of malnutrition	66 (48.9)
	Normal nutritional status	44 (32.6)
Risk for falls according to the Downton Fall Risk Index (n=135)		
	High risk for falls	122 (90.4)
	Low risk for falls	13 (9.6)
Risk for pressure injury according to the Braden scale (n=135)		
	High risk	19 (14.1)
	Moderate risk	17 (12.6)
	Low risk	26 (19.3)
	No risk	73 (54.1)
Level of dementia according to the Clinical Dementia Rating (n=135)		
	Severe dementia	30 (22.2)
	Moderate	22 (16.3)
	Mild dementia	24 (17.8)
	Very mild dementia	31 (23.0)
	Normal	28 (20.7)
Cognitive status according to the Mini-Mental State Examination (n=135)		
	Cognitive impairment	54 (40.0)
	No cognitive impairment	58 (23)
	Refused to respond	23 (17.0)
Cognitive status according to the Semantic Verbal Fluency test (n=135)		
	Cognitive impairment	90 (66.7)
	No cognitive impairment	18 (27)
	Refused to respond	27 (20.0)
Cognitive status according to the Clock Drawing Test (n=135)		
	Cognitive impairment	56 (41.5)
	No cognitive impairment	37 (42)
	Refused to respond	42 (31.1)
Depression according to the Cornell Scale (n=76)		
	No depression	73 (96.1)
	Probable depression	3 (3.9)
Depression according to the Geriatric Depression Scale: Short Form (n=59)		
	No depression	41 (69.5)
	Depression	16 (27.1)
	Severe depression	2 (3.4)
Level of dependence in activities of daily living according to the Katz Index (n=135)		
	Total dependence	63 (46.7)
	Partial dependence	28 (20.7)
	Independent	44 (32.6)
Level of dependence in instrumental activities of daily living according to the Lawton & Brody Index (n=66)		
	Total dependence	29 (43.9)
	Severe dependence	17 (25.8)
	Mild dependence	11 (16.7)
	Mild dependence	7 (10.6)
	Independent	2 (3.0)

**Figure 1:**
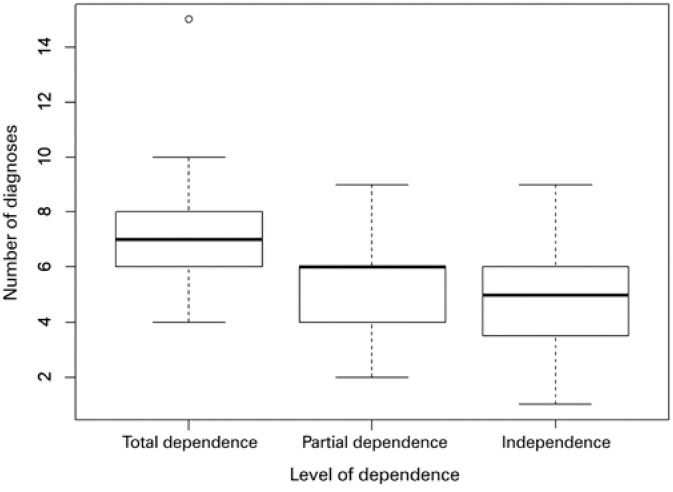
Relationship between the number of nursing diagnoses identified per patient and the level of dependence

There were 52 NDx identified; the number of NDx identified per patient was associated with the level of dependence (Figure 1).

The prevalence of the diagnoses is shown in table 4 The most frequent diagnoses were: risk for falls (00155), risk for impaired skin integrity (00047), risk for pressure ulcer (00249), frail elderly syndrome (00257), overweight (00233), risk for constipation (00015), and obesity (00232).

**Table 4:** Relationship between nursing diagnoses in the elderly residents and the level of dependence according to the Katz Index

Nursing diagnoses		Total (n=135)	Total dependence (n=63)	Partial dependence (n=28)	Independence (n=44)	p value
		n (%)	n (%)	n (%)	n (%)	
Domain 1. Health promotion						
	Frail elderly syndrome	105 (77.8)	63 (100.0)	26 (92.9)	16 (36.4)	<0.001
	Ineffective protection	8 (5.9)	2 (3.2)	2 (7.1)	4 (9.1)	0.195
	Risk-prone health behavior	6 (4.4)	0 (0.0)	2 (7.1)	4 (9.1)	0.021
	Ineffective health management	3 (2.2)	1 (1.6)	1 (3.6)	1 (2.3)	0.779
	Sedentary lifestyle	1 (0.7)	0 (00)	0 (0.0)	1 (2.3)	
Domain 2. Nutrition						
	Overweight	48 (35.6)	23 (36.5)	11 (39.3)	14 (31.8)	0.646
	Obesity	43 (31.9)	12 (19.0)	10 (35.7)	21 (47.7)	0.002
	Impaired swallowing	22 (16.3)	22 (34.9)	0 (0.0)	0 (0.0)	<0.001
	Risk for unstable blood glucose level	21 (15.6)	6 (9.5)	8 (28.6)	7 (15.9)	0.285
	Risk for overweight	8 (5.9)	5 (7.9)	1 (3.6)	2 (4.5)	0.437
	Imbalanced nutrition: less than body requirements	3 (2.2)	2 (3.2)	0 (0.0)	1 (2.3)	0.701
Domain 3. Elimination and exchange						
	Risk for constipation	48 (35.6)	26 (41.3)	9 (32.1)	13 (29.5)	0.202
	Functional urinary incontinence	24 (17.8)	9 (14.3)	6 (21.4)	9 (20.5)	0.387
	Constipation	7 (5.2)	4 (6.3)	1 (3.6)	2 (4.5)	0.654
	Urge urinary incontinence	3 (2.2)	1 (1.6)	1 (3.6)	1 (2.3)	0.779
	Bowel incontinence	2 (1.5)	2 (3.2)	0 (0.0)	0 (0.0)	
	Stress urinary incontinence	2 (1.5)	0 (0.0)	0 (0.0)	2 (4.5)	
	Impaired urinary elimination	2 (1.5)	0 (0.0)	1 (3.6)	1 (2.3)	0.299
Domain 4. Activity/Rest						
	Impaired physical mobility	15 (11.1)	1 (1.6)	1 (3.6)	13 (29.5)	<0.001
	Insomnia	10 (7.4)	8 (12.7)	0 (0.0)	2 (4.5)	0.086
	Risk for decreased cardiac output	3 (2.2)	1 (1.6)	0 (0.0)	2 (4.5)	0.345
	Risk for ineffective peripheral tissue perfusion	3 (2.2)	1 (1.6)	2 (7.1)	0 (0.0)	0.701
	Impaired sleep pattern	2 (1.5)	0 (0.0)	0 (0.0)	2 (4.5)	
	Wandering	2 (1.5)	1 (1.6)	1 (3.6)	0 (0.0)	0.560
	Impaired bed mobility	1 (0.7)	1 (1.6)	0 (0.0)	0 (0.0)	
	Ineffective peripheral tissue perfusion	1 (0.7)	0 (0.0)	1 (3.6)	0 (0.0)	
Domain 5. Perception/cognition						
	Impaired memory	5 (3.7)	0 (0.0)	0 (0.0)	5 (11.4)	
	Impaired verbal communication	5 (3.7)	0 (0.0)	0 (0.0)	5 (11.4)	
Domain 6. Self-perception						
	Low situational self-esteem	1 (0.7)	1 (1.6)	0 (0.0)	0 (0.0)	
Domain 7. Role relationship						
	Dysfunctional family processes	17 (12.6)	3 (4.8)	7 (25.0)	7 (15.9)	0.059
	Interrupted family processes	5 (3.7)	1 (1.6)	0 (0.0)	4 (9.1)	0.055
	Caregiver role strain	3 (2.2)	0 (0.0)	0 (0.0)	3 (6.8)	
	Impaired social interaction	1 (0.7)	0 (0.0)	0 (0.0)	1 (2.3)	
Domain 9. Coping/stress tolerance						
	Anxiety	6 (4.4)	3 (4.8)	1 (3.6)	2 (4.5)	0.941
	Chronic sadness	1 (0.7)	1 (1.6)	0 (0.0)	0 (0.0)	
	Readiness for enhanced resilience	1 (0.7)	0 (0.0)	0 (0.0)	1 (2.3)	
Domain 11. Safety/protection						
	Risk for falls	134 (99.3)	62 (98.4)	28 (100.0)	44 (100.0)	
	Risk for Impaired skin integrity	78 (57.8)	52 (82.5)	14 (50.0)	12 (27.3)	<0.001
	Risk for pressure ulcer	57 (42.2)	49 (77.8)	4 (14.3)	4 (9.1)	<0.001
	Risk for aspiration	39 (28.9)	36 (57.1)	3 (10.7)	0 (0.0)	<0.001
	Risk for Impaired tissue integrity	12 (8.9)	9 (14.3)	3 (10.7)	0 (0.0)	0.012
	Impaired skin integrity	10 (7.4)	3 (4.8)	3 (10.7)	4 (9.1)	0.368
	Risk for Infection	10 (7.4)	9 (14.3)	0 (0.0))	1 (2.3)	0.014
	Impaired tissue integrity	5 (3.7)	3 (4.8)	1 (3.6)	1 (2.3)	0.502
	Risk for Injury	3 (2.2)	3 (4.8)	0 (0.0)	0 (0.0)	
	Risk for other-directed violence	3 (2.2)	1 (1.6)	1 (3.6)	1 (2.3)	0.779
	Risk for bleeding	1 (0.7)	0 (0.0)	0 (0.0)	1 (2.3)	
	Risk for suicide	1 (0.7)	1 (1.6)	0 (0.0)	0 (0.0)	
	Risk for self-mutilation	1 (0.7)	0 (0.0)	1 (3.6)	0 (0.0)	
	Risk for impaired oral mucosa	1 (0.7)	1 (1.6)	0 (0.0)	0 (0.0)	
Domain 12. Comfort						
	Chronic pain	12 (8.9)	2 (3.2)	2 (7.1)	8 (18.2)	0.008
	Acute pain	1 (0.7)	0 (0.0)	0 (0.0)	1 (2.3)	

Eleven NDx were associated with the level of dependence of the resident.

Those associated with dependence were in domains 1 (Health Promotion), 2 (Nutrition) and 11 (Safety/Protection): frail elderly syndrome (00257), impaired swallowing (00103), risk for impaired skin integrity (00047), risk for pressure ulcer (00249), risk for aspiration (00039), risk for impaired tissue integrity (00248), and risk for infection (00004).

Those associated with independence were in domains 1 (Health Promotion), 2 (Nutrition), 4 (Activity/Rest) and 12 (Comfort): risk-prone health behavior (00188), obesity (00232), impaired physical mobility (00085), and chronic pain (00133).

## DISCUSSION

This study identified NDx in elderly residents using data from the clinical history, physical examination, and the administration of instruments validated in the geriatric population. In addition, the NDx were validated by nurses with both clinical and academic experience, and their relationships with the level of dependence in ADL was determined. Expert consensus validations have refined the nursing classifications in order to promote linkages and to collaborate in the definition of better practice standards. The process adopted in our study increases the level of diagnostic accuracy, and allows for the establishment of a diagnostic profile according to the level of dependence.

The level of dependence in ADL in our sample was similar to that identified by other studies in skilled nursing facilities for the elderly, with independence ranging from 32.3 to 41.6%, partial dependence ranging from 15.2 to 51.6%, and total dependence ranging from 16.1 to 43.2%.^(^^5^^,^^6^^)^

The relationship between the greater number of NDx and increased dependence was expected. Specifically, Domains 1 (Health promotion), 2 (Nutrition) and 11 (Safety/Protection).

Most residents in all levels of dependence had risk for falls, in addition to an unsteady gait and the need for mobility devices. Most falls are associated with one or more identifiable risk factors, such as weakness, unsteady gait, confusion, and certain medications. Attention to these risk factors can significantly reduce fall rates. The ideal approach for risk assessment and preventive interventions include collaboration of the multidisciplinary team, with a view toward reducing risk factors.^(^^20^^)^

Risk for impaired skin integrity was found in our study as a frequent nursing diagnosis. The main risk factor for impaired skin integrity was the use of disposable diapers due to urinary incontinence. Diapers are exclusively indicated for those with incontinence, decreased level of consciousness, and severe mobility restrictions. Indiscriminate use can cause skin friction, maceration and dermatitis. In order to avoid skin injuries, diaper changes should be performed as needed, based on the residents’ eliminations, which requires adequate nurse staffing, and professionals who are aware of the risks.^(^^21^^)^

Risk for pressure ulcer was also one of the most prevalent NDx. Both diagnoses, risk for impaired skin integrity and risk for pressure ulcer, were associated with dependence, which was expected, given the risk factors mentioned above. Although they were frequent NDx, impaired skin integrity and impaired tissue integrity were not often identified in our sample, which suggests a positive result of adequate preventive nursing care.

Impaired physical mobility was associated with independence, mainly due to unsteady gait. In the residents that were totally dependent, impaired physical mobility was a defining characteristic of frail elderly syndrome. Even though full recovery of mobility is not possible in all cases, collaborative interdisciplinary work is required so that impaired mobility does not develop into further incapacities.^(^^22^^)^

Both diagnoses of obesity and overweight were associated with independence. Although we did not assess the residents for sarcopenic obesity, it is possible that part of the sample has developed it, given that it is independently associated with and precedes the onset of IADL disability in the elderly.^(^^23^^)^

In addition to those with obesity and overweight, a significant number of residents were underweight or at risk of malnutrition, and 73.3% used nutritional supplements. A previous Brazilian study also found a prevalence of risk for malnutrition of 66.3% in elderly residents.^(^^9^^)^ Therefore, a concept analysis and conceptual development of risk for malnutrition would be of interest, to bring awareness to this concept, especially within the elderly population.

Impaired swallowing also serves as a risk factor for risk for aspiration; both are NDx associated with dependence, and which can influence malnutrition. Thirty to 60% of older adults have been noted to have impaired swallowing, for whom the use of thickeners is indicated, as found in our sample.^(^^24^^)^

Risk-prone health behavior, defined by NANDA-I as “impaired ability to modify lifestyle and/or actions in a manner that improves the level of wellness”,^(^^4^^)^ was mainly evidenced through substance misuse and smoking. Based on our clinical practice, independent older adults are more likely than dependent ones to make choices that directly influence their health in a positive or negative way, such as smoking, using alcohol, and adopting a sedentary lifestyle.

In our sample, a few residents smoked or suffered from alcoholism. A previous study found that 12% of the elderly smoked, and 9.3% used alcohol, out of whom 85.8% did it once to four times a week.^(^^25^^)^ It is relevant that the elderly understand the context regarding their own health, so that they are capable of taking better care of themselves.

Chronic pain was associated with independence, possibly because independent residents are able to report pain more easily. For a more precise diagnosis of pain, one should not only be restricted to the patient's self-report, but should include clinical history, chronic conditions, physical examination, biopsychosocial assessment, allergies, and drug reactions. Pain characteristics, such as frequency, exacerbating and alleviating factors, and functional impact should be included. Multidimensional instruments such as the McGill Pain Questionnaire may be useful.^(^^26^^)^

Frail elderly syndrome was expected to be associated with dependence, given its definition by NANDA-I: Dynamic state of unstable equilibrium that affects the older individual experiencing deterioration in one or more domain of health (physical, functional, psychological, or social) and leads to increased susceptibility to adverse health effects, in particular disability.^(^^4^^)^

A cluster of NDx that occurred together in our sample – thereby consisting of a syndrome – were: impaired walking, feeding self-care *deficit*, toileting self-care *deficit*, dressing self-care *deficit*, impaired memory, and impaired physical mobility. The intervention that best addresses the diagnoses together seems to be physical exercise. A recent meta-analysis found positive impacts of exercise on falls, mobility, balance performance, functional ability, muscle strength, and body composition, but more studies with frail populations are needed to select the most favorable exercise program.^(^^27^^)^

Risk for infection was associated with dependence because this diagnosis was noted in most of the residents with malnutrition, invasive devices (gastrostomy buttons), impaired mobility, limitations in self-care, and fecal/urinary incontinence: all of which favor respiratory, skin, and urinary infections. Therefore, this diagnosis can be prevented by treatment and/or prevention of other related NDx. Studies have described positive effects of habitual physical exercise on the immune system.^(^^28^^)^

Risk for constipation was also frequent in our sample. In order to prevent constipation, walking is recommended for older adults who are fully mobile or who have limited mobility (15-20 minutes once or twice a day; or 30-60 minutes daily or 3 to 5 times per week), at least 50 feet twice a day for those with limited mobility. For those with an unsteady gait or restricted to bed, exercises such as pelvic tilt, low trunk rotation and single leg lifts are recommended. The mean fluid intake in our sample was also below the recommended amount to prevent constipation (1,500-2,000mL/day). The residents should be encouraged to take sips of fluid throughout the day.^(^^29^^)^

Impaired sleep pattern was not frequent, because 75.6% of the sample used sleeping medication to enhance sleep, and the treatment was effective. Some nursing interventions to support an adequate sleep pattern have been found to be effective, such as music, music video watching, listening to natural sounds, back massage, acupuncture, and aromatherapy but the studies have low or very low evidence.^(^^30^^)^

Our results are limited by the initial assessment of a single nurse. However, all experts had access to full residents’ information to support their decisions regarding the NDx. This study supports the assessment of aspects related to the level of dependence in ADL of elderly residents, using multiple validated gerontological scales. Our method can be reproduced in other skilled nursing facilities for the elderly for accurate assessment.

## CONCLUSION

Fifty-two NANDA International nursing diagnoses were identified and validated in elderly residents of a nursing home for the elderly. Eleven of those nursing diagnoses were associated with the level of dependence in activities of daily living. These results allow for the planning of interventions directed at patient-specific nursing diagnoses, rather than directing care at a general category of dependence. Ultimately, this provides for patient-centered nursing care, considering individual patient characteristics, which may better meet the needs of the residents for nursing care.
