# Supplementary material for: Relationships between nursing diagnoses and the level of dependence in activities of daily living of elderly residents
Source: Einstein (Sao Paulo). 2020 Oct 29;18:eAO5445. doi: 10.31744/einstein_journal/2020AO5445 (PMC7607918; doi:10.31744/einstein_journal/2020AO5445)
Supplement: Translation

# Translation: Relações entre diagnósticos de enfermagem e nível de dependência para atividades da vida diária de idosos

**Authors:** Karina Mello Dias, Tracy Heather Herdman, Renata Eloah de Lucena Ferretti-Rebustini, Camila Takao Lopes, Eduarda Ribeiro dos Santos

**Affiliations:** 1 São PauloSP Brasil Faculdade Israelita de Ciências da Saúde Albert Einstein, Hospital Israelita Albert Einstein, São Paulo, SP, Brasil.; 2 Green BayWI United States University of Wisconsin-Green Bay, Green Bay, WI, United States.; 3 São PauloSP Brasil Escola de Enfermagem, Universidade de São Paulo, São Paulo, SP, Brasil.; 4 São PauloSP Brasil Escola Paulista de Enfermagem, Universidade Federal de São Paulo, São Paulo, SP, Brasil.

**Keywords:** Idoso, Enfermagem geriátrica, Instituição de longa permanência para idosos, Diagnóstico de enfermagem, Processo de enfermagem

## Abstract

**Objetivo:**

Identificar e validar diagnósticos de enfermagem em idosos de uma instituição de longa permanência e determinar sua relação com o nível de dependência para atividades da vida diária.

**Métodos:**

Foram avaliados 135 idosos por meio de histórico clínico e exame físico. Utilizaram-se 12 instrumentos gerontológicos validados para avaliar *delirium*, estado nutricional, risco de quedas, risco de lesão por pressão, demência, declínio cognitivo, depressão e nível de dependência para atividades da vida diária e atividades instrumentais da vida diária. Os diagnósticos de enfermagem foram identificados e validados por enfermeiros doutores com *expertise* clínica. A associação entre a presença de diagnóstico de enfermagem e o nível de dependência foi avaliada por teste de tendência de proporções. A associação entre o número de diagnósticos de enfermagem e o nível de dependência dos idosos foi verificada pelo teste de hipótese de Kruskal-Wallis.

**Resultados:**

A maioria dos idosos apresentou risco de desnutrição, alto risco de quedas, declínio cognitivo, dependência total para atividades da vida diária e atividades instrumentais da vida diária e demência muito leve. A maior parte não apresentou risco de lesões por pressão. A depressão foi verificada entre aqueles com demência, mas estava ausente naqueles sem demência. Foram validados 52 diagnósticos de enfermagem. Destes, 11 foram associados ao nível de dependência para atividades da vida diária.

**Conclusão:**

Estes resultados podem ser reproduzidos em outras instalações qualificadas para idosos e permitir o planejamento de intervenções para aliviar etiologias e sinais/sintomas dos diagnósticos de enfermagem, em vez de simplesmente direcionar os cuidados para uma categoria geral de dependência e, assim, garantir cuidados de enfermagem individualizados para atender às necessidades específicas de cada residente.

## INTRODUÇÃO

Em todo o mundo, o envelhecimento da população tem ocorrido de forma acelerada nas últimas décadas. Em 2020, o Instituto Brasileiro de Geografia e Estatística (IBGE) estima a existência de 20.816 milhões de idosos no Brasil, e a expectativa é que esse número dobre até 2044. Além disso, espera-se que a população idosa represente mais de 25% do total da população brasileira em 2060, ultrapassando as estimativas para países desenvolvidos.^(^^1^^)^

Apesar dos avanços tecnológicos que ajudam na prevenção e no tratamento de doenças, a população idosa é marcada pela redução das capacidades funcionais, levando a uma variedade de limitações físicas e psicológicas e dificultando a realização de atividades diá;rias e a manutenção de atividades sociais aceitáveis.^(^^2^^)^

O declínio funcional refere-se à dificuldade ou necessidade de ajuda, para realização de atividades diárias, incluindo atividades da vida diária (AVD) e atividades instrumentais de vida diária (AIVD). Em gerontologia, a avaliação da funcionalidade dos idosos é parâmetro importante das AVD e disponibiliza informação relevante sobre a saúde e as necessidades de apoio dessa população durante a realização das AVD.^(^^2^^)^

Devido ao prolongamento de vida da população, em geral, há necessidade de estabelecer estruturas adequadas para assistência a esses indivíduos, como instituições de longa permanência para idosos (ILPI). Essas instituições requerem uma equipe especializada e multidisciplinar. Os enfermeiros têm papel crucial no cuidado e na educação em saúde dos residentes dessas instituições, com impacto direto na qualidade da assistência prestada.^(^^3^^,^^4^^)^

A partir da perspectiva da enfermagem, identificar a acurácia dos diagnósticos de enfermagem (DE) é essencial, para estabelecer resultados centrados no paciente e planejar intervenções apropriadas. O uso de instrumentos de medida validados é recomendado pela NANDA International, Inc., (NANDA-I) para assegurar a relevância e a acurácia diagnóstica.^(^^4^^)^

Poucos estudos antigos no Brasil foram publicados sobre o processo de enfermagem em ILPI. Contudo, nenhum deles validou seus achados em relação à acurácia, utilizando escalas gerontológicas validadas.^(^^5^^,^^6^^)^

## OBJETIVO

Identificar e validar os diagnósticos de enfermagem em idosos em instituições de longa permanência, e determinar a relação entre os diagnósticos de enfermagem e o nível de dependências desses residentes.

## METÓDOS

### Desenho e local do estudo

Trata-se de estudo analítico e transversal realizado no Residencial Israelita Albert Einstein (RIAE), uma ILPI, localizada na cidade de São Paulo (SP), Brasil, com capacidade para assistência de 170 residentes.

### Participantes

Amostra de conveniência incluindo 135 indivíduos residentes na instituição entre maio e julho de 2016, independentemente do sexo ou idade.

### Coleta de dados

Uma enfermeira especialista em enfermagem geriátrica coletou dados do histórico clínico e realizou exame físico dos residentes. Além disso, utilizou 12 instrumentos gerontológicos validados para investigar *delirium*, es;tado nutricional, risco de quedas, risco de lesão por pressão, demência, estado cognitivo, depressão e nível de dependência para AVD e AIVD.

A tabela 1 identifica todos os instrumentos, o foco das medidas, quem conduziu a avaliação, de quem os dados foram obtidos, as características consideradas pelas ferramentas, como são pontuadas e as referências críticas.

**Tabela 1:** Instrumentos de avaliação geriátrica utilizadas para avaliar os residentes

Nome da escala	Foco da avaliação	Quem conduziu a avaliação	De quem os dados foram obtidos	Características consideradas	Escore
*Confusion Assessment Method for Intensive Care Unit*^(^^7^^)^	*Delirium*	Enfermeira pesquisadora	Idoso residente	1. Início agudo e curso de flutuação dos sintomas de delírio 2. Desatenção 3. Nível de consciência alterado 4. Pensamento desorganizado	Características 1 e 2 presentes e características 3 ou 4 também presentes: ^Confusion Assessment Method for Intensive Care Unit^ positivo, ^delirium^ presente
Mini Avaliação Nutricional^®(8)^	Estado nutricional	Enfermeira pesquisadora	Idoso residente	18 itens, incluindo antropometria, avaliação diária, avaliação clínica global, e autopercepção do estado de saúde e nutricional	24-30 pontos: estado nutricional normal 17-23,5 pontos: risco de desnutrição 17 pontos: desnutrição
Escala de risco de queda de Downton^(^^9^^)^	Risco de queda	Enfermeira pesquisadora	Idoso residente, prontuário e familiar	Histórico de quedas, medicações, ^deficit^ sensorial, estado mental e caminhada	Cada fator pode obter um escore de 1 ponto; escore ≥3 identifica pacientes em risco
Escala de Braden^(^^10^^)^	Risco de lesão por pressão	Enfermeira pesquisadora	Idoso residente	6 subescalas: percepção sensorial, atividade, mobilidade, hidratação, nutrição, fricção ou cisalhamento	Escore total pode variar de 6-23 pontos, e pacientes são classificados como muito alto risco de lesão de pressão (≤9 pontos), alto risco (10-12), risco moderado (13-14), baixo risco (15-18) e sem risco (19-23)
*Clinical Dementia Rating*^(^^11^^)^	Desempenho cognitivo e funcional na demência	Enfermeira pesquisadora e um médico	Idoso residente e/ou familiar	6 domínios: memória, orientação, julgamento e solução de problemas, negócios comunitários, casa e ^hobbies^ e cuidados pessoais	Escala de 5 pontos. Escore geral é calculado por meio do uso de algoritmo: – 0: saudável – 0,5: questionável – 1: demência leve – 2: demência moderada – 3: demência grave
Miniexame do Estado do Mental^(^^12^^)^	Comprometimento cognitivo	Enfermeira pesquisadora	Idoso residente	Questionário de 30 pontos agrupados em 7 categorias, cada uma representando um domínio/função cognitiva diferente: orientação no tempo (5 pontos); orientação no espaço (5 pontos); registro de 3 palavras (3 pontos); atenção e cálculo (5 pontos); lembrança de 3 palavras (3 pontos); linguagem (8 pontos) e construção visual (1 ponto)	As notas de corte para prejuízo cognitivo variam de acordo com a escolaridade: – 18: analfabeto – 21: 1 a 3 anos de escolaridade – 24: 4 a 7 anos de escolaridade – 26: mais de 7 anos de escolaridade
Teste da Fluência Verbal Semântica^(^^13^^)^	Comprometimento cognitivo	Enfermeira pesquisadora	Idoso residente	Meios de capacidade de armazenamento do sistema de memória semântica, habilidade de recuperar informação armazenada na memória e processamento das funções executivas. Esse teste inclui o relato do maior número possível de nomes de animais ao longo de 1 minuto e designa 1 ponto para cada animal nomeado	Resultados são pontuados como: – 9 pontos: analfabeto – 12 pontos: 1-8 anos de estudo – 13 pontos: 9 ou mais anos de estudo
Teste do Desenho do Relógio^(^^14^^)^	Comprometimento cognitivo	Enfermeira pesquisadora	Idoso residente	Os residentes receberam um papel e caneta. tendo sido solicitado que seguissem as instruções: “Primeiro, desenhe um relógio com todos os números. Segundo, coloque as mãos sob o relógio e simule a leitura de 2 horas e 45 minutos”	10-6 pontos: desenho do relógio com círculo e número está geralmente intacto -5- 1 ponto: desenho do relógio e número não está intacto Escores <6 são classificados como comprometimento cognitivo
*Geriatric Depression Scale: Short Form*^(^^15^^)^	Depressão em idosos sem demência	Enfermeira pesquisadora	Idoso residente	Escala de 15 itens, em que a resposta positiva a 10 itens indica presença de depressão, enquanto resposta negativa a 5 itens indica depressão	– 0-5: ausência de depressão – 6-10: depressão leve – 9-11: depressão moderada – 11-15: depressão grave
Escala de Cornell^(^^16^^)^	Depressão em idosos com demência	Enfermeira pesquisadora	Entrevista com o cuidador	19 itens, cada um avaliado pela gravidade, em uma escala de 0-2: – 0: ausência – 1: leve ou intermitente – 2: grave	Escore total ≤9 equivale a ausência de depressão; 10-17 indica depressão provável; 18 está correlacionada à depressão
Índice de Katz^(^^17^^)^	Autonomia para ADV	Enfermeira pesquisadora	Idoso residente, prontuário e familiar	Avaliação por classificação de desempenho adequado no banho, vestir-se, continência, higiene, transferência e alimentação. Em cada atividade, os residentes foram classificados como “sim” e “não” por independência	Classificado como totalmente dependente quando classificado com ≤2 sins foram obtidos; parcialmente dependentes (3 ou 4 pontos) e totalmente dependente (5 ou 6 pontos)
Escala de Lawton e Brody^(^^18^^)^	Independência para AIVD	Enfermeira pesquisadora	Idoso residente e familiar	8 domínios: habilidade do uso do telefone, preparação de refeições, manutenção da casa, lavagem de roupa, modo de transporte, responsabilidade pelos próprios medicamentos e habilidade de lidar com as finanças	Classificação: – 0-1: dependência total – 2-3: dependência grave – 4-5: dependência moderada – 6-7: dependência leve – 8: independência

AVD: atividades da vida diária; AIVD: atividades instrumentais de vida diária.

Após coleta de dados, as hipóteses de DE da NANDA-I foram identificadas por uma enfermeira especializada em geriatria e uma com nível de Doutorado. Após, a identificação das hipóteses diagnósticas foi avaliada por três enfermeiras com expertise clínica, com nível de Doutorado e publicações científicas relacionadas à gerontologia e ao Processo de Enfermagem.

O investigador principal enviou o arquivo com antecedência para as especialistas, contendo dados coletados sobre cada residente. As especialistas se encontraram pessoalmente três vez, por aproximadamente quatro horas, revisaram a descrição de cada paciente e discutiram cada DE de cada residente, até alcançar consenso de 100% quanto à relevância dos DE apoiados pela classificação de diagnósticos da NANDA-I 2015-2017.^(^^19^^)^

### Considerações éticas

Os participantes do estudo ou seus familiares assinaram um Termo de Consentimento, assegurando o anonimato e garantindo a liberdade em participar ou abandonar o estudo, se assim desejado. O protocolo do estudo foi aprovado pelo Comitê de Ética em Pesquisa, protocolo 1.446.621, CAAE: 53671316.4.0000.0071.

### Análise dos dados

Os DE foram analisados descritivamente (frequências absolutas e relativas) de acordo com o nível do Índice de Katz de dependência para AVD. A associação entre a presença de um DE e nível de dependência foi avaliada por meio de teste de tendências para proporções. Para investigar a associação entre o número de DE e o nível de dependência dos idosos, foi usado o teste de hipótese de Kruskal-Wallis. As análises foram realizadas utilizando o *software* R, ou software *SPSS* versão 3.1.3. Considerou-se nível de 5% como significante.

## RESULTADOS

A tabela 2 apresenta o perfil clínico e demográfico dos idosos residentes. Todos eram judeus, com idade variando entre 60 a 102 anos, a maioria mulheres, viúvas e do lar antes de viver na instituição. Os residentes dormiam um número satisfatório de horas por noite (7,65±1,31 horas), apesar de 75,6% (n=102) utilizarem medicamentos para induzir o sono. O tabagismo e o uso de álcool não foram comuns. A média de índice de massa corporal (IMC) foi 27,5±5,5kg/m^2^. Trinta e três residentes (31,9%) eram obesos, 48 (35,6%) tinham sobrepeso, 41 (30,4%) tinham peso adequado e três (2,22%) estavam com baixo peso. O método de alimentação mais comum foi oral, com boa aceitação. Dentre os que eram alimentados por via oral, 20 (15,9%) necessitavam de uso de espessantes. Trinta e seis idosos (26,7%) utilizavam suplementos. A média de consumo de líquidos foi 810±350mL/dia.

**Tabela 2:** Características clínicas e sociodemográficas dos residentes idosos

Variáveis		Média±DP
Idade, anos		85,7±8,0
		n (%)
Sexo		
	Feminino	107 (79,3)
	Masculino	28 (20,7)
Religião: judaismo		135 (100,0)
Estado civil		
	Viúvo	81 (60,0)
	Solteiro	28 (20,7)
	Casado	20 (14,8)
	Divorciado	6 (4,4)
Ocupação antes viver na instituição	
	Do lar	59 (43,7)
	Comerciante	30 (22,2)
	Professor	9 (6,7)
	Secretário	9 (6,7)
	Outros	28 (20,7)
Hábitos		
	Tabagismo	3 (2,2)
	Uso de álcool	2 (1,5)
	Uso de pílulas para dormir	102 (75,6)
Método de alimentação		
	Oral	126 (93,3)
	Gastrostomia	9 (6,7)
Aceitação de alimento		
	Boa	109 (86,5)
	Regular	15 (11,9)
	Ruim	2 (1,6)
Uso de suplementos orais		36 (26,7)
*Deficit* visual		102 (75,6)
Perda de audição		56 (41,5)
Comprometimento da fala		4 (3,0)
Marcha instável		103 (76,3)
Necessidade de dispositivo de mobilidade		
	Cadeira de rodas	64 (45,9)
	Nenhum	34 (26,7)
	Andador	33 (24,4)
	Bengala	4 (3,0)

DP: desvio padrão.

A tabela 3 apresenta as avaliações clínicas baseada em instrumentos gerontológicos validados. A maioria dos residentes estava em risco de desnutrição, tinha algum risco de queda, não tinha risco para lesões por pressão, apresentava demência muito leve, tinha prejuízo cognitivo, cognitiva, depressão entre aqueles com demência, ausência de depressão entre aqueles sem de;mên;cia, dependência total para AVD e AIVD.

**Tabela 3:** Avaliação clínica de residentes idosos por meio de instrumentos validados

Avaliação		n (%)
Estado nutricional de acordo com a Mini Avaliação Nutricional^®^ (n=135)		
	Desnutrição	25 (18,5)
	Risco de desnutrição	66 (48,9)
	Estado nutricional normal	44 (32,6)
Risco de queda baseado na escala de risco de queda de Downton (n=135)		
	Alto risco para quedas	122 (90,4)
	Baixo risco para quedas	13 (9,6)
Risco de lesão por pressão de acordo com escala de Braden (n=135)		
	Alto risco	19 (14,1)
	Risco moderado	17 (12,6)
	Baixo risco	26 (19,3)
	Sem risco	73 (54,1)
Nível de demência de acordo com Clinical Dementia Rating (n=135)		
	Demência grave	30 (22,2)
	Demência moderada	22 (16,3)
	Demência leve	24 (17,8)
	Demência muito leve	31 (23,0)
	Normal	28 (20,7)
Estado cognitivo de acordo com Miniexame do Estado do Mental (n=135)		
	Comprometimento cognitivo	54 (40,0)
	Sem comprometimento cognitivo	58 (23)
	Recusa para responder	23 (17,0)
Estado cognitivo de acordo com Teste da Fluência Verbal Semântica (n=135)		
	Comprometimento cognitivo	90 (66,7)
	Sem comprometimento cognitivo	18 (27)
	Recusa para responder	27 (20,0)
Estado cognitivo de acordo com Teste do Desenho do Relógio (n=135)		
	Comprometimento cognitivo	56 (41,5)
	Sem comprometimento cognitivo	37 (42)
	Recusa para responder	42 (31,1)
Depressão de acordo com escala de Cornell (n=76)		
	Sem depressão	73 (96,1)
	Depressão provável	3 (3,9)
Depressão de acordo com Geriatric Depression Scale: Short Form (n=59)		
	Sem depressão	41 (69,5)
	Depressão	16 (27,1)
	Depressão grave	2 (3,4)
Nível de dependência para atividades da vida diárias de acordo com Índice de Katz (n=135)		
	Dependência total	63 (46,7)
	Dependência parcial	28 (20,7)
	Independência	44 (32,6)
Nível de dependência para atividades instrumentais de vida diária de acordo com índice de Lawton e Brody (n=66)		
	Dependência total	29 (43,9)
	Dependência grave	17 (25,8)
	Dependência leve	11 (16,7)
	Dependência leve	7 (10,6)
	Independência	2 (3,0)

Identificaram-se 52 DE; o número de DE identificados por paciente se associou ao nível de dependência (Figura 1).

**Figura 1:** Relações entre o número de diagnósticos de enfermagem identificados por paciente e nível de dependência

A prevalência de diagnósticos é apresentada na tabela 4. Os diagnósticos mais frequentes foram: risco de quedas (00155), risco de integridade da pele prejudicada (00047), risco de lesão por pressão (00249), síndrome da fragilidade do idoso (00257), sobrepeso (00233), risco de constipação (00015) e obesidade (00232).

**Tabela 4:** Relação entre diagnósticos de enfermagem em idosos residentes e nível de dependência de acordo com escala de Katz

Diagnósticos de enfermagem		Total (n=135) n(%)	Dependência total	Dependência parcial (n=28)	Independência (n=44)	Valor de p
		n(%)	n=63)	n(%)	n(%)	
Domínio 1. Promoção de saúde						
	Síndrome da fragilidade do idoso	105 (77,8)	63 (100,0)	26 (92,9)	16 (36,4)	<0,001
	Proteção ineficaz	8 (5,9)	2 (3,2)	2 (7,1)	4 (9,1)	0,195
	Comportamento de saúde propenso ao risco	6 (4,4)	0 (0,0)	2 (7,1)	4 (9,1)	0,021
	Controle ineficaz da saúde	3 (2,2)	1 (1,6)	1 (3,6)	1 (2,3)	0,779
	Estilo de vida sedentário	1 (0,7)	0 (00)	0 (0,0)	1 (2,3)	
Domínio 2. Nutrição						
	Sobrepeso	48 (35,6)	23 (36,5)	11 (39,3)	14 (31,8)	0,646
	Obesidade	43 (31,9)	12 (19,0)	10 (35,7)	21 (47,7)	0,002
	Deglutição prejudicada	22 (16,3)	22 (34,9)	0 (0,0)	0 (0,0)	<0,001
	Risco de glicemia instável	21 (15,6)	6 (9,5)	8 (28,6)	7 (15,9)	0,285
	Risco de sobrepeso	8 (5,9)	5 (7,9)	1 (3,6)	2 (4,5)	0,437
	Nutrição desiquilibrada: menor do que as necessidades corporais	3 (2,2)	2 (3,2)	0 (0,0)	1 (2,3)	0,701
Domínio 3. Eliminação e troca						
	Risco de constipação	48 (35,6)	26 (41,3)	9 (32,1)	13 (29,5)	0,202
	Incontinência urinária funcional	24 (17,8)	9 (14,3)	6 (21,4)	9 (20,5)	0,387
	Constipação	7 (5,2)	4 (6,3)	1 (3,6)	2 (4,5)	0,654
	Incontinência urinária de urgência	3 (2,2)	1 (1,6)	1 (3,6)	1 (2,3)	0,779
	Incontinência intestinal	2 (1,5)	2 (3,2)	0 (0,0)	0 (0,0)	
	Incontinência urinária por estresse	2 (1,5)	0 (0,0)	0 (0,0)	2 (4,5)	
	Eliminação urinária prejudicada	2 (1,5)	0 (0,0)	1 (3,6)	1 (2,3)	0,299
Domínio 4. Atividade/Repouso						
	Mobilidade física prejudicada	15 (11,1)	1 (1,6)	1 (3,6)	13 (29,5)	<0,001
	Insônia	10 (7,4)	8 (12,7)	0 (0,0)	2 (4,5)	0,086
	Risco de débito cardíaco diminuído	3 (2,2)	1 (1,6)	0 (0,0)	2 (4,5)	0,345
	Risco de perfusão tissular periférica ineficaz	3 (2,2)	1 (1,6)	2 (7,1)	0 (0,0)	0,701
	Padrão do sono prejudicado	2 (1,5)	0 (0,0)	0 (0,0)	2 (4,5)	
	Perambulação	2 (1,5)	1 (1,6)	1 (3,6)	0 (0,0)	0,560
	Mobilidade no leito prejudicada	1 (0,7)	1 (1,6)	0 (0,0)	0 (0,0)	
	Perfusão tissular periférica ineficaz	1 (0,7)	0 (0,0)	1 (3,6)	0 (0,0)	
Domínio 5. Percepção/cognição						
	Memória prejudicada	5 (3,7)	0 (0,0)	0 (0,0)	5 (11,4)	
	Comunicação verbal prejudicada	5 (3,7)	0 (0,0)	0 (0,0)	5 (11,4)	
Domínio 6. Autopercepção						
	Baixa estima situacional	1 (0,7)	1 (1,6)	0 (0,0)	0 (0,0)	
Domínio 7. Papéis e relacionamentos						
	Processos familiares disfuncionais	17 (12,6)	3 (4,8)	7 (25,0)	7 (15,9)	0,059
	Processos familiares interrompidos	5 (3,7)	1 (1,6)	0 (0,0)	4 (9,1)	0,055
	Tensão do papel do cuidador	3 (2,2)	0 (0,0)	0 (0,0)	3 (6,8)	
	Interação social prejudicada	1 (0,7)	0 (0,0)	0 (0,0)	1 (2,3)	
Domínio 9. Enfrentamento/Tolerância ao estresse						
	Ansiedade	6 (4,4)	3 (4,8)	1 (3,6)	2 (4,5)	0,941
	Tristeza crônica	1 (0,7)	1 (1,6)	0 (0,0)	0 (0,0)	
	Disposição para resiliência melhorada	1 (0,7)	0 (0,0)	0 (0,0)	1 (2,3)	
Domínio 11. Segurança/proteção						
	Risco de quedas	134 (99,3)	62 (98,4)	28 (100,0)	44 (100,0)	
	Risco de integridade da pele prejudicada	78 (57,8)	52 (82,5)	14 (50,0)	12 (27,3)	<0,001
	Risco de lesão por pressão	57 (42,2)	49 (77,8)	4 (14,3)	4 (9,1)	<0,001
	Risco de aspiração	39 (28,9)	36 (57,1)	3 (10,7)	0 (0,0)	<0,001
	Risco de integridade tissular prejudicada	12 (8,9)	9 (14,3)	3 (10,7)	0 (0,0)	0,012
	Integridade da pele prejudicada	10 (7,4)	3 (4,8)	3 (10,7)	4 (9,1)	0,368
	Risco de infecção	10 (7,4)	9 (14,3)	0 (0,0))	1 (2,3)	0,014
	Risco de integridade tissular prejudicada	5 (3,7)	3 (4,8)	1 (3,6)	1 (2,3)	0,502
	Risco de lesão	3 (2,2)	3 (4,8)	0 (0,0)	0 (0,0)	
	Risco de violência direcionada a outros	3 (2,2)	1 (1,6)	1 (3,6)	1 (2,3)	0,779
	Risco de sangramento	1 (0,7)	0 (0,0)	0 (0,0)	1 (2,3)	
	Risco de suicídio	1 (0,7)	1 (1,6)	0 (0,0)	0 (0,0)	
	Risco de automutilação	1 (0,7)	0 (0,0)	1 (3,6)	0 (0,0)	
	Risco de mucosa oral prejudicada	1 (0,7)	1 (1,6)	0 (0,0)	0 (0,0)	
Domínio 12. Conforto						
	Dor crônica	12 (8,9)	2 (3,2)	2 (7,1)	8 (18,2)	0,008
	Dor aguda	1 (0,7)	0 (0,0)	0 (0,0)	1 (2,3)	

Onze DE estavam associados ao nível de dependência dos residentes. Os DE associados à dependência estavam nos domínios 1 (Promoção da Saúde), 2 (Nutrição) e 11 (Segurança/Proteção) e eram os seguintes: síndrome da fragilidade do idoso (00257), deglutição prejudicada (00103), risco de lesão na integridade da pele (00047), risco de lesão por pressão (00249), risco de aspiração (00039), risco de integridade tissular prejudicada (00248) e risco de infecção (00004).

Os DE associadas à independência estavam nos domínios 1 (Promoção da Saúde), 2 (Nutrição), 4 (Atividade/Repouso) e 12 (Conforto) e eram os seguintes: comportamento de saúde propenso a risco (00188), obesidade (00232), mobilidade física prejudicada (00085) e dor crônica (00133).

## DISCUSSÃO

Este estudo identificou DE em residentes idosos utilizando dados do histórico clínico, exame físico e aplicação de instrumentos validados para população geriátrica. Além disso, os DE foram validadas por enfermeiros com experiência clínica e acadêmica, e a relação entre os DE e o nível de dependência para AVD dos residentes foi determinada. Validações de consenso de especialistas têm refinado as classificações de enfermagem para promover conexões e colaborar na identificação de padrões de melhores práticas. O processo adotado em nosso estudo aumentou o nível de acurácia diagnóstica e permitiu estabelecimento de perfil diagnóstico de acordo com nível de dependência.

O nível de dependência para AVD em nossa amostra foi similar ao identificado em outros estudos em ILPI, com independência variando de 32,3 a 41,6%, dependência variando de 15,2 a 51,6% e total dependência variando de 16,1 a 43,2%.^(^^5^^,^^6^^)^

A relação entre o grande número da DE e a dependência aumentada era esperada, especialmente, nos domínios 1 (Promoção da Saúde), 2 (Nutrição), e 11 (Segurança/Proteção).

A maioria dos residentes em todos níveis de dependência apresentou risco de quedas, além de marcha instável e necessidade de dispositivos de mobilidade. A maioria das quedas esteva associada a um ou mais fatores de risco identificados, como fraqueza, marcha instável, confusão e a alguns medicamentos. A atenção aos fatores de risco pode reduzir significativamente as taxas de quedas. A abordagem ideal para avaliação de risco e intervenção preventiva incluiu a colaboração da equipe multidisciplinar, com objetivo de reduzir os fatores de risco.^(^^20^^)^

O risco de integridade da pele prejudicada foi frequentemente diagnosticado no estudo. O principal fator de risco para integridade da pele prejudicada foi o uso de fraldas descartáveis, devido à incontinência urinária. Fraldas são exclusivamente indicadas para aqueles com incontinência, redução do nível de consciência e restrições de mobilidade aguda. O uso indiscriminado pode causar fricção da pele, maceração e dermatite. Para evitar lesões cutâneas, a trocas das fraldas devem ser realizadas regularmente com base nas evacuações dos residentes, o que requer quantidade adequada de profissionais de enfermagem, e profissionais que estejam cientes sobre os riscos.^(^^21^^)^

O risco de lesão por pressão também foi um dos DE mais prevalentes. Ambos os DE, risco de integridade da pele prejudicada e risco de lesão por pressão, estavam associados à dependência, algo esperado, dados os fatores de risco. Apesar de serem DE frequentes, integridade da pele prejudicada e integridade tissular prejudicada não foram comumente identificados em nossa amostra, o que sugere resultado positivo de assistência de enfermagem preventiva adequada.

Mobilidade física prejudicada esteve associada à independência, principalmente devido à marcha instável. Em residentes que eram totalmente dependentes, a mobilidade física prejudicada foi definida como uma característica de síndrome do idoso frágil. Apesar de a total recuperação da mobilidade não ser possível em todos os casos, um trabalho interdisciplinar colaborativo é necessário, assim a mobilidade prejudicada não evolui para outras incapacidades.^(^^22^^)^

Ambos os diagnósticos de obesidade e sobrepeso foram associados à independência. Apesar de os residentes não terem sido avaliados quanto à obesidade sarcopênica, é possível que parte da amostra a tenha desenvolvido, dado que se associa de maneira inde;pendente e precede o início do distúrbio das AIVD em idosos.^(^^23^^)^

Além dos residentes com obesidade e sobrepeso, número significativo apresentava sobrepeso ou risco de desnutrição, e 73,3% utilizavam suplementos nutricionais. Um estudo brasileiro anterior também encontrou prevalência de risco para desnutrição de 66,3% em residentes idosos.^(^^9^^)^ Portanto, uma análise e o desenvolvimento conceitual do risco de desnutrição seriam interessantes para promover conscientização a respeito deste conceito, especialmente na população de idosos.

Deglutição prejudicada também é um fator de risco para risco de aspiração, e ambos são DE associados à dependência e podem influenciar na desnutrição. A deglutição prejudicada tem sido identificada em 30% a 60% dos idosos, sendo, para estes, indicado o uso de espessantes, como observado em nossa amostra.^(^^24^^)^

O comportamento de saúde propenso ao risco, definido pelo NANDA-I como “capacidade prejudicada de modificar o estilo de vida e/ou as ações de forma a melhorar o nível de bem-estar”^(^^4^^)^ foi principalmente evidenciado por meio do uso indevido de substâncias e tabagismo. Baseado em nossa prática clínica, idosos independentes são mais propensos, do que os dependentes, a escolher o que influenciará diretamente em sua saúde de forma negativa ou positiva, tais como tabagismo, álcool e estilo de vida sedentário.

Em nossa amostra, poucos residentes eram tabagistas ou alcoolistas. Um estudo anterior encontrou que 12% dos idosos eram fumantes, e 9,3% consumiam álcool, dos quais 85,8% o faziam uma a quatro vezes por semana.^(^^25^^)^ É relevante que os idosos entendam o contexto em relação à sua saúde, para que sejam capazes de se cuidar de forma mais adequada.

A dor crônica foi associada à independência, possivelmente porque os residentes independentes são capazes de relatar a dor mais facilmente. Para um diagnóstico de dor mais preciso, não se deve apenas restringir-se ao autorrelato dos pacientes, mas incluir histórico clínico, condições crônicas, exame físico, avaliação biopsicossocial, alergias e reações medicamentosas. As características da dor, como frequência, exacerbação e fatores de alívio e impacto funcional, devem ser incluídas. Instrumentos multidimensionais, como o questionário de dor de McGill, podem ser úteis.^(^^26^^)^

Esperava-se que a síndrome do idoso frágil fosse associada à dependência, dada sua definição pela NANDA-I: Estado dinâmico de equilíbrio instável que afeta o idoso que passa por deterioração em um ou mais de um domínio de saúde (físico, funcional, psicológico ou social) e leva a aumento da suscetibilidade a efeitos de saúde adversos, em particular, a incapacidade.^(^^4^^)^

Um grupo de DE que ocorreu concomitantemente em nossa amostra, consistindo em uma síndrome, foi: deambulação prejudicada, *deficit* no autocuidado para alimentação, *deficit* no autocuidado para banho, *deficit* no autocuidado para vestir-se, memória prejudicada e mobilidade física prejudicada. A intervenção que melhor aborda os DE em conjunto parece ser o exercício físico. Uma metanálise encontrou impactos positivos do exercício físico na ocorrência de quedas, mobilidade, equilíbrio, habilidade funcional, força muscular e composição corporal. Contudo, estudos adicionais com populações frágeis são necessários para selecionar programas de exercícios mais favoráveis.^(^^27^^)^

O risco de infecção se associou à dependência, pois esse DE foi identificado na maioria dos residentes com desnutrição, dispositivos invasivos (botões de gastrostomia), mobilidade prejudicada, limitações para autocuidado e incontinência fecal/urinária: todos favorecem infecções respiratórias, de pele e urinárias. Portanto, o diagnóstico pode ser prevenido por meio de tratamento e/ou prevenção de outros DE relacionados. Estudos têm descrito efeitos positivos do hábito de exercício físico no sistema imune.^(^^28^^)^

O risco de constipação também foi mais frequente em nossa amostra. Para prevenir constipação em idosos com mobilidade preservada ou que têm mobilidade limitada, recomendam-se exercícios de 14 a 20 minutos uma ou duas vezes, ou 30 a 60 minutos diariamente ou três a cinco vezes por semana, ou pelo menos 50 passos duas vezes por dia para aqueles com mobilidade limitada. Para aqueles com marcha instável ou restritos ao leito, exercícios como elevação pélvica, rotação do tronco, elevação única das pernas são recomendados. A média de ingestão de líquidos também esteve abaixo do total recomendado para prevenir constipação (1.500 a 2.000mL/dia). Os residentes devem ser incentivados a ingerir pequenas quantidades de líquido durante o dia.^(^^29^^)^

O padrão de sono prejudicado não foi frequente, devido a 75,6% da amostra utilizarem indutores do sono e pela efetividade do tratamento. Algumas intervenções de enfermagem para apoiar um padrão de sono adequado têm sido efetivas, tais como ouvir música, assistir a vídeos de música, ouvir sons da natureza, massagens nas costas, acupuntura e aromaterapia. Contudo essas práticas têm pouca ou muito pouca evidência científica.^(^^30^^)^

Nossos resultados são limitados, considerando que a coleta de dados foi conduzida por uma única enfermeira. Porém, todas as especialistas tiveram acesso às informações completas dos residentes para apoiar as decisões em relação aos DE. Nosso estudo apoia a avaliação de aspectos relacionados ao nível de dependência para AVD entre idosos residentes em ILPI por meio de múltiplas escalas gerontológicas validadas. Nosso método deve ser reproduzido em outras ILPI para obter uma avaliação mais acurada.

## CONCLUSÃO

Foram identificados evalidados entre os idosos de uma instituição de saúde de longa permanência 52 diagnósticos de enfermagem da NANDA-I. Destes, 11 diagnósticos de enfermagem estiveram associados ao nível de dependência para atividade de vida diária. Esses resultados permitem planejar intervenções diretas para diagnósticos de enfermagem específicos para os pacientes, ao invés de uma assistência à saúde direcionada a uma categoria geral de dependência. Por fim, tais resultados também permitem um cuidado centrado no paciente, levando em conta as características indivi;duais de cada paciente para melhor atendimento às necessidades dos idosos residentes em relação aos cuidados de enfermagem.
